# Increased seroprevalence of HAV and parvovirus B19 in children and of HEV in adults at diagnosis of autoimmune hepatitis

**DOI:** 10.1038/s41598-018-35882-7

**Published:** 2018-11-28

**Authors:** Richard Taubert, Jana Diestelhorst, Norman Junge, Martha M. Kirstein, Sven Pischke, Arndt Vogel, Heike Bantel, Ulrich Baumann, Michael P. Manns, Heiner Wedemeyer, Elmar Jaeckel

**Affiliations:** 1 Department of Gastroenterology, Hepatology and Endocrinology, Hannover Medical School, Hannover, Germany; 2 Pediatric Gastroenterology and Hepatology, Department of Pediatric Kidney, Liver and Metabolic Diseases, Hannover Medical School, Hannover, Germany; 3 German Center for Infection Research (DZIF), Partner Site Hannover-Braunschweig, Braunschweig, Germany; 4 Present Address: Department of Internal Medicine, Center for Internal Medicine, University Medical Center Hamburg-Eppendorf, Hamburg, Germany; 5 Present Address: Department of Gastroenterology and Hepatology, Essen University Hospital, University of Duisburg-Essen, Essen, Germany

## Abstract

Preceding viral infections have mostly been described in autoimmune hepatitis (AIH) in single cases. We aimed to identify viral infections that potentially trigger AIH, as suggested for hepatitis E virus (HEV) infections. Therefore, antibodies against hepatitis A (HAV), B, C and E viruses; hepatotropic herpesviruses; and parvovirus B19 (PVB19) were analyzed retrospectively in 219 AIH patients at diagnosis, 356 patients with other liver diseases and 89 children from our center. Untreated adult AIH (aAIH) patients showed higher anti-HEV seroprevalences at diagnosis than patients with other liver diseases. Untreated aAIH patients had no increased incidence of previous hepatitis A, B or C. Antibodies against hepatotropic herpesviruses in untreated AIH were in the range published for the normal population. Untreated pediatric AIH (pAIH) patients had evidence of more previous HAV and PVB19 infections than local age-matched controls. The genetic AIH risk factor HLA DRB1*03:01 was more frequent in younger patients, and DRB1*04:01 was more frequent in middle-aged patients without an obvious link to virus seropositivities. Pediatric and adult AIH seem to be distinct in terms of genetic risk factors and preceding viral infections. While associations cannot prove causal relations, the results suggest that hepatotropic virus infections could be involved in AIH pathogenesis.

## Introduction

Autoimmune hepatitis (AIH) is an immune-mediated liver disease that affects all age groups with an increasing incidence and prevalence^1^. Animal models, in which danger signals and genetic predispositions are both necessary to induce AIH, have strengthened the hypothesis of an externally triggered breach of tolerance in genetically predisposed individuals^2–4^.

The only human genetic risk factors that could be confirmed by a large multicenter genome-wide association study were MHC class II molecules^5^. Environmental factors such as drugs and preceding viral infections, which have been suggested to act as external triggers of AIH, were much more diverse^6^. While it can be difficult to discriminate between autoimmune-like drug-induced hepatitis and drug-induced liver injury, the difficulty with viral triggers is the subclinical course of multiple virus infections and the varying time lapse between the infection and diagnosis of AIH. Nevertheless, associations between hepatotropic herpesviruses and hepatitis A (HAV) and C (HCV) viruses have been described over many years but mostly on the basis of case reports, the sole breach of humoral tolerance or sequence similarities between viruses and human molecules^7–10^.

We have recently observed a higher seroprevalence of anti-HEV antibodies in AIH than in other chronic liver and autoimmune diseases in the first large cross-sectional analysis of potential viral triggers of adult AIH^11^. However, an increased incidence of anti-HEV antibodies in the AIH population compared to the normal population could not be confirmed in a multicenter study in the neighboring Netherlands^12^. Both studies could potentially be biased by (I) not focusing on the time of AIH diagnosis but rather testing at any time and (II) by not excluding overlap syndromes with other autoimmune liver diseases. Beyond this, only one pediatric cohort has been analyzed for antibodies against the herpes simplex virus (HSV) and the hepatitis C virus (HCV)^10^.

Thus, we want to assess the hypothesis that previous hepatotropic viral infections predispose patients to a break of hepatic immune tolerance. Therefore, the present study used a comprehensive approach by determining the prevalence of antibodies against specific hepatitis viruses, hepatotropic herpesviruses and parvovirus B19 (PVB19) in pediatric and adult AIH patients at the time of diagnosis before the start of an AIH-specific treatment.

## Results

Our study represents the largest cohort of untreated patients with AIH having serum samples exclusively at the time of diagnosis. This is important because seroprevalence for common viral infections increases with age. This large cohort enabled us to investigate seroprevalence in age-matched groups.

### Age-dependent prevalence of HEV antibodies

**Figure 1:**
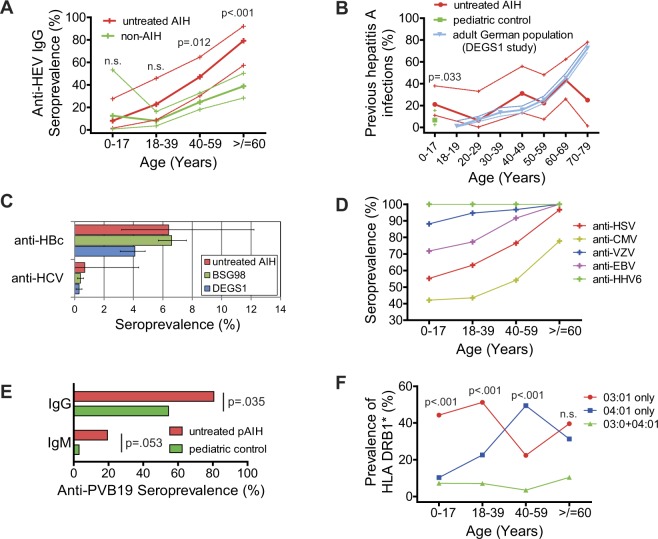
Age-dependent prevalence of viral seroprevalence and genetic risk factors. (**A**) Hepatitis E virus seroprevalence in untreated AIH and non-AIH liver diseases (patient numbers for all serological tests are outlined in Table 1 and Suppl. Tables 1–3). Significant results from the age-dependent Fisher’s exact tests are depicted in the graphs. The dotted lines represent 95% confidence intervals (CIs). (**B**) Prevalence of previous hepatitis A infections in untreated AIH patients, in the German population from a health monitoring study (DEGS1)^43^ and in a local pediatric control either without liver diseases or with non-AIH liver diseases. The dotted lines represent CIs. (**C**) Seroprevalence in untreated adult AIH patients and in the adult German population according to two health monitoring studies (BSG98, DEGS1)^14^. The error bars represent the CIs. (**D**) Seroprevalence of anti-HSV, anti-VZV, anti-CMV, anti-EBV, and anti-HHV6 in patients with untreated AIH. (**E**) Prevalence of anti-parvovirus B19 (PVB19) antibodies in patients with untreated pediatric AIH and in a local pediatric control (Table 1). (**F**) Prevalence of HLA DRB1 risk alleles in AIH patients without overlap syndromes (0–17 years: n = 97; 18–39 years: n = 84; 40–59 years: n = 85; ≥60 years: n = 48) based on our previous studies^26,27^. Only p-values of the comparison of 03:01 against 04:01 are depicted (n.s. = not significant).

Anti-HEV antibodies were detectable in 42/105 (40.0%) pediatric and adult patients with untreated AIH and in 75/322 (23.3%) in pediatric (n = 8) and adult (n = 314) patients with non-AIH liver disease with an age-dependent increase in prevalence (Table 1, Fig. 1A, Suppl. Table 1). In untreated AIH patients diagnosed at 40 years and older, anti-HEV IgG antibodies were significantly more often detected compared to non-AIH liver diseases.

**Table 1:** Data of patients with available serology for HEV, HAV and PVB19.

	total	anti-HEV				anti-HAV				anti-PVB19	
	untreated AIH	untreated AIH		non-AIH liver disease		untreated AIH		local pediatric control	DEGS1 study*	untreated AIH pediatric	local pediatric control
		pediatric	adult	pediatric	adult	pediatric	adult				
Numbers	219	25	80	8	314	56	135	87	6585	31	33
female gender (%)	72%	76%	65%	25%	52%	75%	69%	44%	53%	84%	52%
age (median (IQR) in years)	40.5 (42.9)	14.7 (3.4)	49.9 (24.1)	13.3 (4.8)	50.5 (21.7)	13.0 (6.1)	53.2 (22.5)	12.7 (5.0)	n.d.	12.9 (4.3)	12.0 (6.4)
**Age distribution**											
0–17	71	25		8		56		87		31	33
18–39	36		22		88		33		1692		
40–59	60		34		146		54		2509		
>/=60	52		24		80		48		2384		

*Unadjusted numbers from DEGS1 study^14,43^; n.d.: not determined.

Thereby, prevalences of anti-HEV antibodies were quite similar, when non-AIH liver diseases were separated into the respective entities (Suppl. Fig. 1, Suppl. Table 2). In patients with AIH/primary sclerosing cholangitis (PSC) overlap syndromes, anti-HEV IgG antibodies were not detectable (0/10; 0.0%). This prevalence was lower than that in untreated AIH patients (Suppl. Fig. 1; p = 0.013) and was not different from that in PSC patients (8/75 (10.4%); p = 0.588). In contrast, anti-HEV IgG antibodies were detectable in 13/26 (50.0%) patients with AIH/primary biliary cholangitis (PBC) overlap and showed an age-dependent increase, as in AIH and PBC patients (21/80 (26.3%)). Overall, the antibody prevalence was higher than that in PBC patients (Suppl. Fig. 1; p = 0.037) but was similar to that in untreated aAIH patients (p = 1.0).

HEV RNA was not detectable in 135/144 patients with anti-HEV IgG seropositivity, while 9/144 patients could not be tested due to insufficient sample volume. For HEV-IgM antibodies 29 anti-HEV-IgG positive and 29 anti-HEV-IgG negative AIH patients have been tested, only one of them, an anti-HEV-IgG positive patient, was positive for anti-HEV-IgM, but at same time negative for HEV RNA.

Apart from older age and marginally higher alkaline phosphatase levels, anti-HEV-IgG-positive patients with untreated AIH were not significantly different from seronegative patients in terms of baseline parameters (Table 2).

**Table 2:** Comparison of anti-HEV IgG-positive and -negative untreated AIH patients.

AIH (all age groups)	Anti-HEV IgG		Anti-HEV IgG		p value
	neg	n	pos	n	
Age at diagnosis (years)	26.4 (39.9)	63	56.6 (16.1)	42	<0.001
Gender (male/female)	20/43		13/29		1.000
**Autoantibodies**					
ANA	8/62		7/42		0.585
SMA	8/63		11/41		0.076
LKM	2/62		0/41		0.516
SLA	2/30		0/14		1.000
pANCA	11/27		3/6		1.000
**Laboratory test**					
IgG (times ULN)	1.4 (0.9)	62	1.5 (0.9)	41	0.407
Alanine aminotransferase (times ULN)	21.4 (22.4)	61	16.6 (25.7)	40	0.317
Aspartate aminotransferase (times ULN)	18.3 (26.2)	61	17.2 (23.3)	40	0.634
Alkaline phosphatase (times ULN)	1.1 (0.5)	61	1.5 (1.1)	40	0.049
Bilirubin (times ULN)	3.2 (15.0)	61	3.3 (9.0)	39	0.992
Prothrombin time (%)	68.0 (32.0)	60	70.5 (36.0)	40	0.620
**Histology**					
mHAI	9.0 (6.0)	52	9.0 (2.0)	38	0.827
Fibrosis (Ishak)	2.0 (3.0)	54	3.0 (4.0)	38	0.235

### Age-dependent prevalence of antibodies against hepatitis viruses in untreated AIH

Anti-HAV IgG antibodies were found in 83/191 (43.5%) untreated pediatric and adult AIH patients (Table 1). To differentiate between vaccination and previous hepatitis A, the vaccination status of 78% of IgG-positive patients could be retrieved. The number of anti-HAV-IgG-negative patients was adjusted for the availability of the vaccination status. The rate of previous HAV infections (anti-HAV IgG positivity without vaccination) of the untreated aAIH patients was not different from that of the adult German population (n = 6585; Table 1), as recorded in a recent health monitoring study (DEGS1) using the same Architect (Abbott) test platform^13,14^, because the confidence intervals from both cohorts overlapped broadly (Fig. 1B).

In the respective pediatric German health monitoring study (KIGGS; n = 13063), no information on HAV vaccination status was available^15^. Thus, a local pediatric comparator cohort consisting of children without evidence of liver or autoimmune diseases (n = 34), who mostly exhibited abdominal discomfort or stool abnormalities, and with non-AIH liver diseases (n = 53) was identified, and the vaccination status was retrieved similar to that of pAIH patients (Table 1). Compared to this local pediatric control cohort, untreated pAIH patients had significantly more previous HAV infections (IgG-positive without previous vaccination) (Fig. 1B, Suppl. Table 1). This age matched local control cohort did not exhibit a female preponderance (Table 1). However, HAV infections were similarly found in men and women^14^.

Pediatric AIH patients with previous hepatitis A were older at the time of diagnosis and had a lower prothrombin level compared to those without previous HAV infections (Table 3).

**Table 3:** Comparison of untreated pediatric AIH patients with and without previous hepatitis A.

Pediatric AIH (0–17 years)	No previous		Previous		p value
	hepatitis A	n	hepatitis A	n	
**Age at diagnosis (years)**	12.8 (6.6)	43	16.1 (3.9)	11	**0.005**
Gender (male/female)	11/32		1/10		0.421
**Autoantibodies**					
ANA	34/43		10/11		0.667
SMA	36/43		8/11		0.408
LKM	4/42		1/10		1.000
SLA	2/41		1/10		0.488
pANCA	24/42		5/10		0.734
**Laboratory test**					
IgG (times ULN)	1.7 (1.0)	42	1.6 (1.6)	11	0.405
Alanine aminotransferase (times ULN)	15.1 (21.1)	43	16.1 (29.3)	11	0.562
Aspartate aminotransferase (times ULN)	13.5 (23.3)	43	15.2 (34.1)	11	0.472
Alkaline phosphatase (times ULN)	1.0 (0.8)	43	1.0 (0.3)	11	0.863
Bilirubin (times ULN)	1.8 (4.9)	42	6.6 (19.8)	11	0.087
Prothrombin time (%)	72.0 (21.0)	42	59.0 (26.0)	11	0.026
**Histology**					
mHAI	8.0 (5.3)	38	8.0 (5.5)	11	0.662
Fibrosis (Ishak)	3.0 (2.3)	37	2.0 (2.5)	11	0.544

Additionally, anti-HBc and anti-HCV IgG antibodies were detectable in 9/140 (6.4%) patients and in 1/145 (0.7%) untreated aAIH patients, and an age-dependent increase in the anti-HBc antibody prevalence was observed. Anti-HBc-positive aAIH patients were either anti-HBs-positive (8/9) or anti-HBe-positive (1/9). Compared to the normal German population (the two health monitoring studies, DEGS1 2008–2011 and BSG98 1997–1999) anti-HBc (aAIH: 6.4%, CI: 3.2–12.2; BSG98: 6.6%, CI: 5.7–7.6; DEGS1: 4.1%, CI: 3.1–4.8) and anti-HCV (aAIH: 0.69%, CI: 0.03–4.36; BSG98: 0.4%, CI: 0.2–0.6; DEGS1: 0.3%, CI: 0.1–0.5) were not different in untreated aAIH patients (Fig. 1C, Suppl. Tables 1 and 3)^14^. Most children with untreated pAIH have been tested for anti-HBc and anti-HCV IgG antibodies from the referring medical caregiver already. Thus, only twelve were tested directly in our center and all of them were seronegative.

### Age-dependent prevalence of antibodies against other hepatotropic viruses

The prevalence of IgG antibodies against hepatotropic herpesviruses was in the range described for the normal population (Fig. 1D; Suppl. Table 3)^16–24^. Eleven patients were positive for IgM antibodies (6x HSV, 3x HHV6, 2x VZV), but each showed IgG positivity against the respective herpesvirus. Thus, a distinction between recent primary infection and reactivation was not possible.

Anti-parvovirus B19 (PVB19) IgG antibodies were determined in 31 of 71 treatment-naïve pAIH patients (Table 1). Thereby, 25/31 (80.6%; CI: 61.9–91.9) pAIH patients were seropositive; in comparison, the seroprevalence was found to be 45–50% in children in the German population^15,18,25^. To exclude inter-assay variations with published reports, anti-PVB19 IgG was assessed in all available samples of our local pediatric control with or without non-AIH liver diseases, similar to HAV above. Using these tests, the seroprevalence in untreated pAIH patients was found to be significantly higher than in this pediatric control (18/33; 54.5%; CI:36.6–71.5) (Fig. 1E, Table 1, Suppl. Table 1). Six of 31 (19.4%; CI: 8.1–38.1) pAIH patients who were tested for PVB19 had an anti-PVB19-IgM antibody in addition to anti-PVB19 IgG positivity, and in three of these, active replication was excluded based on a PCR analysis of their blood. The IgM prevalence showed a non-significant trend to be higher in pAIH patients compared to pediatric controls (1/32; 3.1%; CI: 0.2–18.0) (Fig. 1E).

Untreated pAIH patients with anti-PVB19 IgG seropositivity had higher frequencies of anti-nuclear antibodies (ANA) compared to seronegative patients (Table 4). Additionally, there was no evidence for a relevant selection bias between those with PVB19 serology and those without (Table 4).

**Table 4:** Comparison of untreated pediatric AIH patients with and without serology for Parvovirus B19.

Pediatric AIH (0–17 years)	not tested (A)	n	Anti-PVB19 IgG		Anti-PVB19		p value (multi-group comparison A-C)	p value (B versus C)
			negative (B)	n	IgG pos (C)	n		
Age at diagnosis (years)	13.3 (7.2)	40	12.0 (8.5)	6	12.9 (4.3)	25	0.925	0.789
Gender (male/female)	10/30		0/6		5/20		0.370	0.553
**Autoantibodies**								
ANA	32/40		3/6		23/25		0.053	0.038
SMA	11/40		4/6		23/25		0.129	0.159
LKM	6/39		2/6		1/25		0.121	0.088
SLA	4/37		0/6		2/24		0.685	1.000
pANCA	20/40		2/6		12/23		0.706	0.651
**Laboratory test**								
IgG (times ULN)	1.7 (0.6)	40	1.4 (0.7)	6	1.9 (1.6)	24	0.271	0.251
Alanine aminotransferase (times ULN)	10.1 (14.9)	40	7.9 (9.8)	6	16.1 (23.8)	25	0.239	0.227
Aspartate aminotransferase (times ULN)	11.4 (19.8)	40	8.4 (15.3)	6	20.4 (30.0)	25	0.296	0.339
Alkaline phosphatase (times ULN)	1.0 (0.5)	40	1.0 (2.6)	6	1.0 (0.4)	25	0.904	0.789
Bilirubin (times ULN)	1.1 (3.4)	39	2.0 (35.0)	6	2.7 (6.4)	25	0.569	0.679
Prothrombin time (%)	71.0 (22.0)	39	72.5 (29.0)	6	69.0 (50.0)	25	0.950	0.981
Haemoglobin (g/dl)	12.4 (3.0)	34	13.2 (0.9)	5	11.8 (3.4)	23	0.546	0.318
**Histology**								
mHAI	7.0 (6.0)	31	7.0 (2.5)	5	9.0 (6.0)	23	0.349	0.380
Fibrosis (Ishak)	3.0 (3.5)	31	4.0 (3.5)	5	3.0 (2.0)	23	0.434	0.560

### Age-dependent prevalence of genetic risk factors

In an age-based reanalysis of our recent studies^26,27^, HLA DRB1*03:01 (single positive or homogenous) was the dominant genotype in children and young adults (0–17 years (n = 97): p < 0.001; n = 97; 18–39 years (n = 84): p < 0.001); the DRB1*04:01 (single positive or homogenous) genotype was most prominent in the middle adulthood age range (40–59 years (n = 85): p < 0.001), and both risk alleles (each single positive or homogenous) were equally present in the late adulthood age range (≥60 years (n = 48): p = 0.522) in AIH without overlap syndromes (Fig. 1F). In AIH/PBC and AIH/PSC overlap syndromes, the patient numbers were too low (n = 30) for an age-dependent analysis.

**Figure 2:**
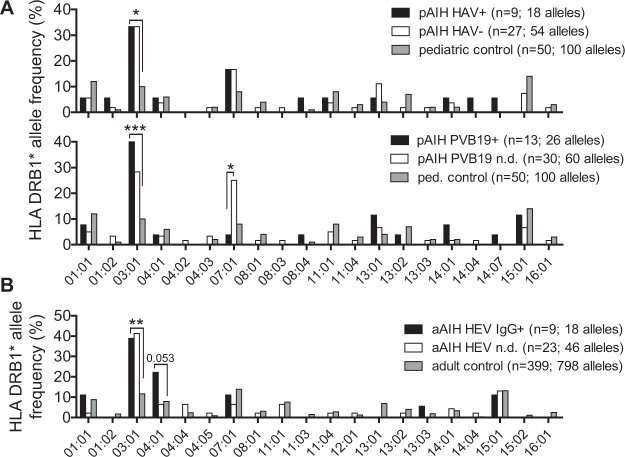
Frequency of HLA DRB1 alleles with regard to virus serology. (**A**) Distribution of HLA DRB1 alleles of pAIH patients (top panel) with (HAV+) and without (HAV-) previous HAV infections or (bottom panel) with anti-PVB19 IgG positivity and those in which these antibodies were not determined (n = 25) or were negative (n = 5; n.d.) compared to the pediatric control from our previous study^26^. (**B**) Patients with aAIH with anti-HEV IgG positivity and those in which these antibodies were not determined (n = 23) or were negative (n = 5; n.d.) compared to an adult control from our previous study^27^ (*p < 0.05; **p < 0.01).

The HLA DRB1* risk allele 03:01 was increased in pAIH patients irrespective of viral seroprevalences (Fig. 2). There were no significant differences in the HLA DRB1* allele frequencies between pAIH patients with and without previous hepatitis A or between anti-HEV IgG positive aAIH patients and the combined comparator group of seronegative ones and those without anti-HEV test. The frequency of the AIH-2 risk allele 07:01 was significantly lower in PVB19 IgG-positive pAIH compared to the combined group of seronegative pAIH patients and those without anti-PVB19 test. However, anti-PVB19 seropositive children and healthy controls had similar frequencies of the allele 07:01 (Fig. 2A).

### Environmental factors shortly preceding the AIH diagnosis

Environmental factors preceding the AIH diagnosis were identified in 11 patients (Suppl. Table 4). One AIH patient was diagnosed 2–3 months after an acute HAV infection without persistent HAV replication. One patient reported the onset of hepatitis symptoms 3 days after an influenza vaccination. The diagnosis of AIH was finally made after 3 months. In six patients of this cohort, the diagnosis of AIH was related to the intake of new drugs (levofloxacin, clarithromycin, an antibiotic agent that could not be remembered, chemotherapy) or drugs that have been described as potential inducers of AIH (simvastatin, infliximab)^6^.

## Discussion

To our knowledge, this is the first comprehensive analysis of previous viral infections and known genetic risk factors over all age groups in the so far largest cohort of exclusively untreated AIH patients.

Recent similarly sized studies analyzed anti-HEV antibodies at any time point in aAIH patients; e.g., months to years after the diagnosis^11,12^. This can cause multiple biases by e.g. viral infections after the diagnosis of AIH, higher infection risk due to more medical procedures and blood transfusions^28^, or a decay of antibody titers under immunosuppression. The current approach increased the differences between AIH and controls from an approximately 1.5 times (33% vs. 21%)^11^ to an approximately 2 times higher age-dependent antibody seroprevalence.

To address pathophysiological associations of previous viral infections and hepatic autoimmunity, we focused exclusively on the earliest possible time point at the diagnosis of AIH. Thus, we could confirm our results and those of others^11,29^ showing a higher anti-HEV antibody prevalence in aAIH patients. We can only speculate whether testing at diagnosis and excluding overlap syndromes would have relevantly changed the results of the Dutch retrospective multicenter study^12^, which found no increased seroprevalence of anti-HEV in AIH patients compared to the population. Although anti-HEV seroprevalences exhibit large regional differences^30^, the Netherlands and the German federal state of Lower Saxony, from where the majority of our patients came, are neighbors. Thus, the age-dependent seroprevalence of the normal population in the Netherlands^12^ and in our non-AIH controls are more or less similar. Furthermore, recent reports^12,29^ and our study applied the same Wantai assay. However, severities of AIH manifestations in our and the Vienna transplant center (median aminotransferase levels were 15–20 times the upper limit of normal (ULN)) were higher than in the Dutch study (10 times ULN). Therefore, we might look at a more severe manifestations. In addition, the significantly higher prevalence in our adult AIH cohort was just 20–40% higher than that in the control group. This means that HEV infections precede diagnosis only in a subgroup of aAIH patients, while other environmental factors, such as drug exposure or altered self-antigen levels could be potential triggers in the remaining patients.

Nonetheless, HEV infections are associated with multiple immune pathologies, mostly of the nervous system, as well as extrahepatic autoimmune diseases^31^. A recent study could demonstrate that humoral tolerance was broken in half of the patients with an acute HEV infection as exemplified by the appearance of autoantibody that are found in AIH as well^32^. However, the anti-HEV seroprevalence in the autoimmune biliary diseases PSC and PBC was lower and in the range of chronic viral hepatitis. This is in accordance with previous and smaller studies in PBC^33,34^ and with the pathophysiological concept that a hepatocellular virus replication more likely triggers an autoimmune response against hepatocytes, as in AIH^35^.

Regarding previous HAV infections, information about the vaccination status is crucial. Although this information was retrievable for 78% of anti-HAV IgG-positive patients, the vaccination status was only documented in the adult but not the pediatric health monitoring study (Dr. Poethko-Müller: personal communication)^13^. Although several case reports have described a close relation between HAV and the onset of AIH^8,10^, this is the first report of a significant association in a larger cohort. Hitherto, protracted HAV infection in Argentine children was only associated with a HLA risk allele for pAIH^36^, but not with the disease onset itself.

A similarly sized study of mostly untreated pAIH patients found a higher prevalence of antibodies against HSV in pAIH patients than in healthy controls^10^. Unfortunately, available sample volumes of pediatric controls were too little for such a direct comparison of all viruses In addition, multiple case reports have described AIH manifestations after hepatotropic viral infection with, e.g., EBV, HSV, and HHV6^10^. This underlies that a lack of an association of viral infections with AIH on a cohort basis does not necessarily mean that an acute infection with a given virus might not be linked to the development of AIH in individual patients.

To our knowledge, this is the first report about a higher seroprevalence of anti-PVB19 antibodies in pAIH. Due to low sample numbers confirmation from other centers is needed. However, PVB19 infections are associated with multiple systemic and organ specific autoimmune diseases and with the appearance of multiple autoantibodies including ANA. Furthermore, PVB19 is also found in liver tissue and is associated with acute hepatitis and liver failure^37^.

The age-dependence of the predisposition of the various HLA II alleles, especially of the HLA-DRB1 locus, is a well-described phenomenon in AIH^26,27,38^. However, this is the first report analyzing HLA DRB1 alleles and preceding viral infections together. Thereby, the major AIH HLA DRB1 risk allele 03:01 was also enriched in patients with preceding HAV, HEV and PVB19. Interestingly, anti-PVB19 seropositivity seem to be associated with a lower prevalence of 07:01 suggesting an association rather with AIH-1 than with AIH-2.

In our emAIH model, adenoviral hepatitis preceded AIH manifestation, with a lag of several months^2^. The positive anti-PVB19-IgM antibodies found in one fifths of these IgG positive patients suggest similar short intervals after PVB19, as has been shown for EBV^9^. Another relevant issue is, whether PVB19 persisted, as has been described^37^, in the liver for a longer period to prime an autoimmune response, as discussed for protracted HAV infections^36^. Studies in type 1 diabetes have clearly shown that the temporal link between an infection and the break of self-tolerance can suggest a potential causative relation^39^. On the other hand, we know from type 1 diabetes that viral triggers are rather linked to the break of tolerance, which can occur years before disease onset. The high proportion of patients with fibrosis at the time of AIH diagnosis argues for a longer period between infection and diagnosis.

Four of nine patients with an environmental trigger shortly preceding the diagnosis of AIH in our cohort (Suppl. Table 4) also had a history of one of the three potential viral risk factors described here. This suggests the possibility of a stepwise predisposition to hepatic autoimmunity resulting from an individual sequence of external triggers.

Nonetheless, this study has obvious limitations; e.g. not all patients were tested for all viruses at the same time and the patient numbers became small when virus serology and HLA genotypes were combined. A further fundamental limitation of such retrospective studies is that statistical associations do not prove a cause-effect relationship. However, the results of this study could not be validated prospectively, because the majority of HAV, HEV and PVB19 infections have a subclinical or very unspecific disease course. Only prospective and longitudinal population-based biorepositories could overcome this limitation, as has been performed for type 1 diabetes in Scandinavia. However, these biorepositories are not available for the vast majority of countries.

A further bias of serological studies like the present one is that false positive results might occur due to polyclonal hypergammaglobulinemia in untreated AIH patients. However, significant differences between AIH patients and control cohorts were only detectable in a minority of viruses and only in certain age groups. Furthermore, there was no significant difference in IgG levels between AIH patients with and without anti-virus antibodies (Tables 2–4).

Viral infections have been described to precede other organ-specific autoimmune diseases, including diabetes, multiple sclerosis and rheumatoid arthritis, and seem to sensitize people towards food antigens in celiac disease^40,41^. The results of the current study strengthen the hypothesis that hepatotropic infections might contribute to the breach of tolerance at least in a subgroup of genetically susceptible individuals. However, AIH is a heterogeneous disease and has no single causative environmental risk factor. Nonetheless, the current study suggests that pediatric and adult AIH are distinct with regard to potential viral triggers as this has been described for genetic risk factors.

## Patients and Methods

### Patients with AIH

We included all available adult and pediatric patients with treatment-naïve and biopsy-proven AIH with no replicative viral hepatitis and an AIH score ≥10^42^ between 1994 and 2017 in whom viral serology was tested at the time of diagnosis at our center. Patients with treatment-naïve pAIH (<18 years at diagnosis) were recruited as described recently^26^. AIH patients with features of overlap syndromes were assigned to AIH/PBC or AIH/PSC, and treated patients were not excluded as the groups would otherwise have been too small for statistical analysis. HLA genotypes of pAIH and aAIH (≥18 years at diagnosis) were taken from our previous studies and were combined in an age-related analysis to look for associations with viral serology or the development of AIH^26,27^. If possible, available retained samples from our clinical laboratories were used for additional serological virus test (further details are mentioned below).

### Comparator cohorts

Data from two large health monitoring studies (KIGGS and DEGS1) were contributed by the Robert Koch Institute (Berlin, Germany). The HAV serology results of 13063 children from the KIGSS study, conducted in 2003–2006^15^, were compared to those of our pAIH cohort in a matched age range from 3–17 years. The HAV serology and vaccination data of 6585 adults from the DEGS1 study, which was conducted in 2008–2011^43^, were utilized in this study. The prevalence of anti-HBV and anti-HCV antibodies of the DEGS1 study was retrieved from previous publications^13,14^.

For all other viruses no such population-based comparator cohorts tested with the same assay were available. Therefore, local comparator cohorts from our clinics were identified. Comparator cohorts of adult non-AIH liver diseases consisted of PSC, PBC and chronic viral hepatitis with hepatitis B, or C (HBV, HCV) were recruited retrospectively between 2012 and 2016. The pediatric cohorts of non-AIH liver disease (non-AIH; non-alcoholic fatty liver disease (n = 27), PSC (n = 19) and alpha 1-antitrypsin deficiency (n = 4), cryptogenic hepatitis (n = 4), toxic hepatitis (n = 1)) and controls without evidence of liver or autoimmune diseases (n = 34) were retrospectively recruited between 2003 and 2016. The characteristics of the patient cohorts are summarized in Table 1 and Suppl. Tables 2 and 3. Vaccination for hepatitis A was determined by telephone interview (mostly in adults) or based on vaccination cards (majority of children).

### Selection strategy for comparator cohorts

Retrospective studies like the current one are likely biased by the limited availability of crucial data (here the virus serology) only in sub-cohorts that are biased, because clinicians performed additional tests only in e.g. atypical or more severe presentations. Further bias may arise from insufficient comparator cohorts.

We addressed the first source of bias by extensive additional serological analyses of available retained samples and by comparisons of seropositive with seronegative AIH patients. To address the second source of bias, we selected comparator cohorts as objective as possible as far as they were available. The best comparators were the health monitoring studies of the German population (DEGS1 (n = 6585 adults)^43^ and KIGSS (n = 13063 children)^15^) that applied the same test assays as our center. These were available for HAV, HBV and HCV. However, the HAV vaccination status was only documented in the adult heath monitoring study.

The next best comparator would be a local comparator cohort representative for the local population. This was not available, neither for adults nor for children. Thus, we selected a local comparator cohort of patients from our adult and pediatric gastroenterology and hepatology clinics (non-AIH liver diseases). The priority for additional serological virus tests in the available retained samples from our clinical laboratories were assigned by the likelihood of showing significant differences between untreated AIH and the best available comparator. This likelihood was estimated from the differences between untreated AIH and available or published comparators and the available number of untested retained samples. Priorities were assigned in descending order: HEV (in adults), HAV, and PVB19 (both in children). So, all available samples with missing virus serology were retested as long as sufficient sample volumes were available.

Because of the low seroprevalence of HEV antibodies in children the available sample numbers were underpowered to demonstrate significant differences for anti-HEV seroprevalence in children. Thus, we did not perform additional anti-HEV tests in comparator cohorts and only included the already available serological data from children with untreated AIH and non-AIH liver diseases. Likewise, serological testing for herpesviruses was not performed, because of low expected differences of anti-herpesvirus seroprevalences between AIH and the comparators.

### Serological analyzes

The majority of serological test results were generated throughout the diagnostic process of AIH and other liver diseases (HAV, HBV, HCV, herpesviruses, PVB19 in AIH, HEV in non-AIH liver diseases). Additional serological tests were performed from retained samples from our clinical laboratories that were cryo-conserved at −20 °C immediately after the clinical work up.

The testing for the presence of anti-HEV IgG and IgM as well as for HEV RNA was performed as described previously^11^. In the present study, anti-HEV IgG was detected exclusively using the Wantai assay (Wantai, Bejing). Borderline test results, which were generally rare, were considered as positive in all analyses. Anti-HEV IgG testing from AIH patients diagnosed before 2012, the introduction of the Wantai assay in our laboratories, was performed from these retained samples. Anti-IgM and HEV-RNA testing was only performed, when sufficient sample volumes were available after the test for anti-HEV IgG.

The testing for hepatitis A (HAV), HBV and HCV were performed using standardized routine clinical assays on the Architect platform (Abbott Laboratories, Chicago, Illinois); testing for anti-cytomegalovirus (CMV), anti-varicella zoster virus (VZV), anti-herpes simplex virus (HSV), anti-Epstein-Barr virus (EBV), anti-human herpes virus type 6 (HHV6) and anti-parvovirus B19 (PVB19) was performed in the local Department of Virology with standardized routine clinical assays. Additional testing for this cohort was performed using current routine clinical assays: anti-HAV IgG (HAVABlgG2, Abbott) and anti-PVB19 IgG and IgM antibodies (Parv-G and Parv-M assays from Diasorin, Sallugia, Italy).

### Statistical analysis

Statistical analysis was performed using SPSS 15.0 and GraphPad Prism 5. The Mann-Whitney U test was used to compare quantitative data between two groups and the Kruskal-Wallis test for more than two groups. The Fisher’s exact test was used to prepare contingency tables with two groups and Chi² test for more than two groups. P-values below 0.05 (two-tailed) were considered significant in all analyses. Data from the KIGGS and DEGS1 study were analyzed using STATASE 14 software. Confidence intervals of the HAV serology results and previous HAV infections from both studies and our AIH cohorts were compared without further statistical testing.

### Ethics

Written informed consent was obtained from all aAIH patients and from the parents of all pAIH patients. Either the use of clinical routine data from local comparator cohorts and the use of pediatric non-liver and non-AIH controls were approved by informed consent or the need for written informed consent was waived by the institutional review board according to our guidelines. The use of retained samples from our clinical laboratories was approved by the local ethical committee. No research was conducted outside our country. The study conforms to the ethical guidelines of the 1975 Declaration of Helsinki as reflected in a priori approval by the institution’s human research committee. All experiments were performed in accordance with relevant guidelines and regulations. This study was approved by the local research Ethics Committee of Hannover Medical School.

## Electronic supplementary material

Supplementary Material

## Data Availability

All data generated or analysed during this study are included in this published article (and its Supplementary Information files).
