# Supplementary material for: Increased seroprevalence of HAV and parvovirus B19 in children and of HEV in adults at diagnosis of autoimmune hepatitis
Source: Sci Rep. 2018 Nov 28;8:17452. doi: 10.1038/s41598-018-35882-7 (PMC6261942; doi:10.1038/s41598-018-35882-7)
Supplement: Supplementary file 1 — Supplementary Material [file 41598_2018_35882_MOESM1_ESM.pdf]

Increased seroprevalence of HAV and parvovirus B19 in children and of HEV in adults at diagnosis of autoimmune hepatitis.

Richard Taubert[1], Jana Diestelhorst[1,2], Norman Junge[2], Martha M. Kirstein[1], Sven Pischke[1#], Arndt Vogel[1], Heike Bantel[1], Ulrich Baumann[2], Michael P. Manns[1], Heiner Wedemeyer[1,3&], Elmar Jaeckel[1]

[1] Department of Gastroenterology, Hepatology and Endocrinology, Hannover Medical School, Hannover, Germany

[2] Pediatric Gastroenterology and Hepatology, Department of Pediatric Kidney, Liver and Metabolic Diseases, Hannover Medical School, Hannover, Germany

[3] German Center for Infection Research (DZIF), Partner Site Hannover-Braunschweig, Braunschweig, Germany

# current address: Department of Internal Medicine, Center for Internal Medicine, University Medical Center Hamburg-Eppendorf, Hamburg, Germany

& current address: Dept. of Gastroenterology and Hepatology; University Hospital Essen, Essen, Germany

Supplemental Table 1: Summary of viral serology in untreated AIH and comparator groups.

IgG	Patient cohort	Age groups									
		0-17		18-39		40-59		≥ 60		≥ 18	
		seroprevalence	n	seroprevalence	n	seroprevalence	n	seroprevalence	n	seroprevalence	n
anti-HAV without vaccination	untreated AIH	21,9%	56*	8,7%	33*	27,0%	54*	32,3%	48*		
	local pediatric control of non-AIH liver dieseases and non-liver diseases	6,5%	87*								
	DEGS1[#]			1,2-13,6%	1692	16,0-25,2%	2509	45,2-72,6%	2384		6585
anti-HBc	untreated AIH	0,0%	12	2,9%	34	5,3%	57	10,2%	49	6,4%	140
	BSG98 Ref									6,6%	6747
	DEGS1									4,1%	7047
anti-HCV	untreated AIH	0,0%	12	0,0%	35	1,7%	59	0,0%	51	0,7%	145
	BSG98									0,4%	6747
	DEGS1									0,3%	7047
anti-HEV	untreated AIH	8,0%	25	22,7%	22	47,1%	34	79,2%	24		
	local control of non-AIH liver diseases	7,1%	6	8,0%	88	24,7%	146	38,8%	80		
anti-PVB19	untreated AIH	80,6%	31	n.d.		n.d.		n.d.			
	local pediatric control of non-AIH liver dieseases and non-liver diseases	54,5%	33								
anti-CMV	untreated AIH	42,1%	12	43,5%	35	54,1%	59	77,8%	51		
anti-EBV	untreated AIH	71,8%	39	77,3%	22	91,7%	36	100,0%	34		
anti-HHV6	untreated AIH	100,0%	19	100,0%	1	100,0%	6	100,0%	6		
anti-HSV	untreated AIH	55,2%	29	63,2%	19	76,5%	34	96,7%	30		
anti-VZV	untreated AIH	88,2%	17	94,7%	19	96,8%	31	100,0%	29		

* based on these patient numbers with vaccination information in 78% cases (see method section); # data was provided in 10 years intervals, n.d. =not determined

Supplemental Table 2: Data of patients with available anti-HEV testing.

	untreated AIH total	untreated AIH pediatric	untreated AIH adult	AIH/ PSC	AIH/ PBC	PSC	PBC	chronic viral hepatitis	Pediatric non-AIH liver disease
Number	105	25	80	10	26	75	80	165	8
female gender (%)	68%	76%	65%	68%	96.2	30.7	88.8	43.6	25%
age (median (IQR) in years)	45.0 (38.1)	14.7 (3.4)	49.9 (24.1)	25.3 (34.4)	52.4 (13.9)	37.1 (22.8)	54.0 (15.9)	52.9 (21.6)	13.3 (4.8)
Age distribution 0-17	25	25	0	1	0	6	0	0	8
18-39	22	0	22	5	2	37	7	44	0
40-59	34	0	34	3	17	25	45	76	0
>/=60	24	0	24	1	7	7	28	45	0

Supplemental Table 3: Data of patients with available testing for HBV, HCV and herpesviruses.

	untreated AIH total	anti-HBc	anti-HCV	anti-CMV	anti-HSV	anti-VZV	anti-EBV	anti-HHV6
Number	219	152	157	134	112	96	131	32
female gender (%)	72%	71%	71%	70%	71%	71%	70%	81%
age (median (IQR) in years)	40.5 (42.9)	49.8 (27.8)	50.5 (26.9)	44.5 (43.7)	45.7 (43.7)	48.4 (35.6)	44.2 (43.7)	16.2 (38.1)
Age distribution								
0-17	71	12	12	38	29	17	39	19
18-39	36	34	35	23	19	19	22	1
40-59	60	57	59	37	34	31	36	6
>/=60	52	49	51	36	30	29	34	6

Supplemental Table 4: AIH patients with putative direct external triggers.

	AIH manifestation associated with	anti-HEV IgG	previous HAV infection	anti-PVB19 IgG
Drugs	Simvastatin	n.d.	no	pos.
	Infliximab	neg.	no	n.d.
	Chemotherapy for breast cancer	n.d.	no	n.d.
	Levofloxacin	neg.	no	n.d.
	Antibiotics (unknown) but already cirrhosis	n.d.	no	n.d.
	Clarithromycin	n.d.	n.d.	n.d.
	Flue vaccination	pos.	yes	n.d.
Virus infection	EBV seroconversion within previous 34 months	neg.	yes	n.d.
	EBV seroconversion within previous 7 years	neg.	no	pos.
	HEV infection (IgM pos., PCR neg.)	pos.	no	n.d.
	HAV infection 2-3 month before	pos.	yes	pos.

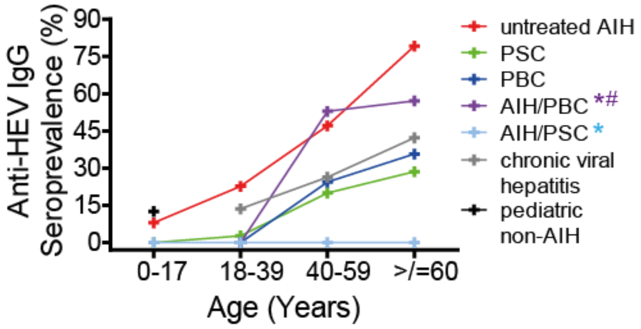

Supplemental Figure 1: Age-dependent prevalence of anti-HEV antibodies

Hepatitis E virus seroprevalence in chronic liver diseases (numbers are as outlined in Supplemental Table 2). The asterisks beside the figure legend depict significant differences from comparisons over all ages of the respective disease groups (#: AIH/PBC compared to PBC). (* and #: $p<0.05$).

References

1 Poethko-Muller, C. *et al.* [Epidemiology of hepatitis A, B, and C among adults in Germany: results of the German Health Interview and Examination Survey for Adults (DEGS1)]. *Bundesgesundheitsblatt Gesundheitsforschung Gesundheitsschutz* **56**, 707-715, doi:10.1007/s00103-013-1673-x (2013).
